# Updating Fearful Memories with Extinction Training during Reconsolidation: A Human Study Using Auditory Aversive Stimuli

**DOI:** 10.1371/journal.pone.0038849

**Published:** 2012-06-29

**Authors:** Javiera P. Oyarzún, Diana Lopez-Barroso, Lluís Fuentemilla, David Cucurell, Carmen Pedraza, Antoni Rodriguez-Fornells, Ruth de Diego-Balaguer

**Affiliations:** 1 Cognition and Brain Plasticity Group, Bellvitge Biomedical Research Institute (IDIBELL), Barcelona, Spain; 2 Department of Basic Psychology, University of Barcelona, Barcelona, Spain; 3 Department of Psychobiology, Faculty of Psychology, University of Málaga, Málaga, Spain; 4 Catalan Institution for Research and Advanced Studies (ICREA), Barcelona, Spain; University of Manchester, United Kingdom

## Abstract

Learning to fear danger in the environment is essential to survival, but dysregulation of the fear system is at the core of many anxiety disorders. As a consequence, a great interest has emerged in developing strategies for suppressing fear memories in maladaptive cases. Recent research has focused in the process of reconsolidation where memories become labile after being retrieved. In a behavioral manipulation, Schiller et al., (2010) reported that extinction training, administrated during memory reconsolidation, could erase fear responses. The implications of this study are crucial for the possible treatment of anxiety disorders without the administration of drugs. However, attempts to replicate this effect by other groups have been so far unsuccessful. We sought out to reproduce Schiller et al., (2010) findings in a different fear conditioning paradigm based on auditory aversive stimuli instead of electric shock. Following a within-subject design, participants were conditioned to two different sounds and skin conductance response (SCR) was recorded as a measure of fear. Our results demonstrated that only the conditioned stimulus that was reminded 10 minutes before extinction training did not reinstate a fear response after a reminder trial consisting of the presentation of the unconditioned stimuli. For the first time, we replicated Schiller et al., (2010) behavioral manipulation and extended it to an auditory fear conditioning paradigm.

## Introduction

Learning to fear is critical for human survival because it impels us to quickly recognize and avoid stimuli that could represent a threat to our lives [1]. In contrast, the modification of this emotional response when circumstances change to safe is equally relevant. The persistence of fearful response in the absence of danger can lead to disabling psychopathology. Today we know that an impaired regulation of fear is the core of many anxiety disorders like Post-Traumatic Stress Disorder (PTSD) [2], phobias and panic disorder [3]. This is the reason why a great amount of effort has been devoted during the last decades to understand the brain mechanisms and neural systems underlying the acquisition of fearful memories and most importantly the way these memories could be successfully modified.

One well described model to study experimentally the acquisition and consolidation of fear memories is the Pavlovian fear conditioning paradigm [4], [5]. In this paradigm an initially neutral stimulus (the conditioned stimulus CS) is repeatedly paired with a biologically aversive event (the unconditioned stimulus US). As the CS-US relation is learned, CS acquires the affective properties of the US generating physiological and behavioural responses such as an increased skin conductance and heart rate responses in humans [6]. One way to counteract such associations is through repeated exposure to the conditioned stimulus in the absence of the aversive outcome or in the case of PTSD patients, presenting reminders of the traumatic event within a safe environment [7], [8], a manipulation called “Extinction training” [9], [10].

However, often fear is recovered spontaneously after the passage of time (spontaneous recovery) [11], after presenting the US alone (reinstatement) [12] or by placing the subject in a context different from the one it was extinguished (renewal) [13]. It is well documented that this recovery of fear occurs because extinction training does not erase fear memories, but instead it generates a new safe memory that would temporarily inhibit the original fear association [14], [15]. In the case of PTSD, since a stressful event augments the noradrenergic activity [16], the consolidation of a traumatic association is highly strengthened by the action of this catecholamine into the amygdala [17]–[19], in consequence this memory prevails over the safe memory that had been previously consolidated through extinction training. This overconsolidation of fear might well be at the root of the high rates of relapse in PTSD patients treated with extinction based therapies [20].

Nowadays, the most promising approach to modify memories that contribute to anxiety disorders is interfering with the reconsolidation of the fear memory. Many studies in rodents have put in evidence that the mere retrieval of a memory triggers a reconsolidation process, during which the memory becomes labile and is vulnerable to modification [21], [22]. The evolutionary advantage of reconsolidation is that the original memory can be reinforced and updated with new relevant information if circumstances change at the time of being retrieved [23].

There is a vast literature in rats reporting erasing of fear by targeting the amygdala with protein synthesis inhibitors after memory retrieval [24]. However, the translation of reconsolidation blockade into humans has been scarcely reported with only one study in patient population [25]. Considering that protein synthesis inhibitors are not a viable technique in humans, researches have used a systemic administration of β-adrenergic receptor antagonist (i.e. propanolol) prior to memory retrieval with encouraging results [26]–[28]. Yet, overall the evidence is still not conclusive [29], [30]. In addition, there are some methodological issues regarding the effects that propanolol might exert over fear responses measures (i.e. Skin Conductance Response and Fear Potentiated Startle Response). In some studies [26]–[28], the habituation to the noise burst (for the fear potentiated startle probes) in the experimental group is conducted while subjects are on propanolol. This might induce a stronger habituation to the startle probe in the drug-reactivation group and thus might explain their lower Fear Potentiated Startle Responses. Moreover, it is necessary to point out that this kind of drugs may not be safe for everyone and may not work equally well in every person [31].

Avoiding the above limitations, a new standpoint drug free behavioral manipulation has been proposed. Two studies, the first carried out in rats and its later follow-up in humans [32], [33], reported that extinction training after memory reactivation leads to a reconsolidation of the fearful association as safe. Capitalizing on reconsolidation as a natural update mechanism, these studies demonstrated that new safe information could be incorporated in the original fearful memory changing its emotional properties permanently. In fact, Monfils et al. [32] showed in fear conditioned rats that when a reminder trial (i.e. single presentation of the CS) is presented before extinction training, a different molecular mechanism in the lateral amygdala is triggered leading to memory destabilization, from when only extinction training is applied. This molecular mechanism has been reported also by Clem and Huganir [34] and Rao-Ruiz et al. [35].

**Figure 1:**
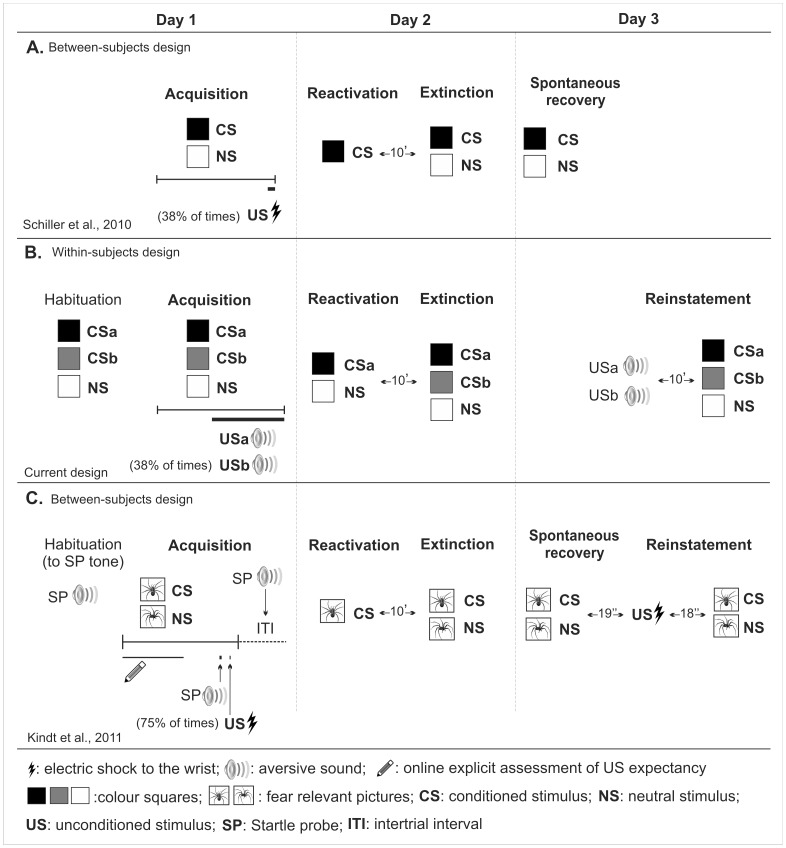
Comparative Experimental Designs of Schiller et al., (2010), current experiment and Kindt et al., (2011). Note that our current design (B) uses a different aversive stimulus modality (sounds instead of electric shocks). It uses a within-subject design and includes a habituation phase for all stimuli. Note that design C (Kind et al., 2011) uses additional measures of fear such as: fear potentiated startle responses and online ratings of US expectancy (in every experimental phase). Design C also uses higher percentage of CS-US pairing, fear relevant pictures instead of colour squares and includes three tests of fear recovery on Day 3 (reinstatement, spontaneous recovery and re-acquisition, last one not shown in the figure). Note also that design C inserts startle probes during CS and NS presentations and during intertrial interval in every experimental phase.

In the human study [33], Schiller et al. (2010; see Fig. 1A) fear conditioned their participants using neutral visual stimuli as the conditioned stimuli (CS) and an electric shock to the wrist as the unconditioned stimuli (US). Fear responses were measured by recording the skin conductance response (SCR). On the following day, in a between-subjects design, participants were assigned to one of three groups: one group where fear memory was reactivated 10 min before extinction training, one where reactivation of the fear memory was performed six hours before extinction training or one where fear memory was not reactivated before extinction training. Twenty-four hours later, spontaneous fear recovery was assessed by receiving re-extinction training. They found that only the group that received extinction training 10 minutes after memory reactivation, thus within the reconsolidation window, did not show recovery of fear. The implication of Schiller et al. [33] study is considered a breakthrough from a clinical standpoint since it provides an exciting possibility for the development of non-invasive treatments for several anxiety disorders.

However, these findings have lacked support by other research groups. Furthermore, the studies that addressed this issue encountered discrepant results in humans [28], [36] and in rats [37]. In humans, using a similar experimental design, Soeter et al. [28] and Kindt et al. [36] failed to replicate Schiller et al. [33] paradigm, first in a within-subject design [28] and later on in a between-subject design [36] (see Fig. 1C). The scantiness of studies and discrepancies has put in a standstill this valuable behavioral manipulation.

Given the crucial utility of this behavioral manipulation, and since it has not been yet successfully replicated by any other research group, a replication of the reported effects is of the utmost importance. Consequently, the objective of our study was to reproduce Schiller et al. [33] findings in a modified version of the paradigm using a different aversive stimulus, in this case auditory. This was done with the purpose of testing if the reported erasing effects would extend to different aversive stimuli. In addition, we applied a within-subjects design because this type of designs requires fewer participants and they are statistically more powerful due to the reduction in error variance associated with individual differences (see experimental scheme in Fig. 1B). The conditioned stimuli were visually presented, as in the Schiller et al. [33] paradigm, but the aversive outcomes were two different sounds instead of one electric shock (see the comparison of the different designs in Fig. 1A and 1B). In addition, the introduction of a different US for each specific CS allowed us to increase the CS-US specificity in order to prevent a single US from recalling the memory of both CS during reactivation.

We found that only the SCR for the conditioned stimulus that was retrieved before extinction training remained extinguished after reinstatement. Supporting Schiller et al. [33] previous findings, our results put on evidence that extinction training within the reconsolidation window can target fearful memories preventing reinstatement of fear.

## Results

In this experiment, for all the analyses, Skin Conductance Responses (SCR) to each of the non-reinforced conditioned and neutral stimuli was used as an index of fear as in Schiller et al. [33]. The experimental design is detailed in Table 1 and follows the 3 days experimental protocol implemented in Schiller et al. (2010): acquisition (Day 1), extinction (Day 2) and re-extinction (Day 3) (Fig. 1B).

**Table 1:** Experimental Design and Timeline.

Day 1	Day 2	Day3
Habituation	Memory Reactivation	Reinstatement
6 CSa	1 CSa	4 USa
6 CSb	1 NS	4 USb
6 NS	***10 min break***	***10 min break***
**Acquisition**	**Extinction**	**Re-Extinction**
10 CSa/6 CSa+USa	10 CSa	10 CSa
10 CSb/6 CSb+USb	10 CSb	10 CSb
10 NS	10 NS	10 NS

CSa: conditioned stimulus a; CSb: conditioned stimulus b; NS: neutral stimulus;

USa: unconditioned stimulus a (sound); USb: unconditioned stimulus b (sound).

On day 1 participants were first habituated to stimuli, immediately afterwards Acquisiton started. On day 2, participants reactivated memory of CSa and NS by one single presentation. After ten minutes, participants underwent extinction training. On day 3, participants were exposed to the aversive sounds. After ten minutes participants underwent Re-Extinction.

First, given that we used two different unconditioned stimuli (USa and USb), a Repeated Measures Analyses of Variance (ANOVA) was performed on Day 1 using Stimuli (CSa, CSb and NS) as a within-subject factor and Sound (USa and USb) as a between-subject factor. Here, we found no significant interaction between Sound and Stimuli (*F* <1*)* confirming that both sounds (USa and USb) generated similar levels of conditioning.

Then, a repeated measures ANOVA was performed with Day (Day 1, Day 2, Day 3) and Stimulus (CSa, CSb, NS) as within subject factors. We found a main effect for Stimuli (*F* (2, 32) = 4.69, *p* = .016) and a trend for Day (*F* (2, 32) = 2.87, *p* = .072). Most importantly, the analysis showed a significant interaction effect between Day and Stimuli (*F* (4, 64) = 4.08, *p* = .005).

### Acquisition

**Figure 2:**
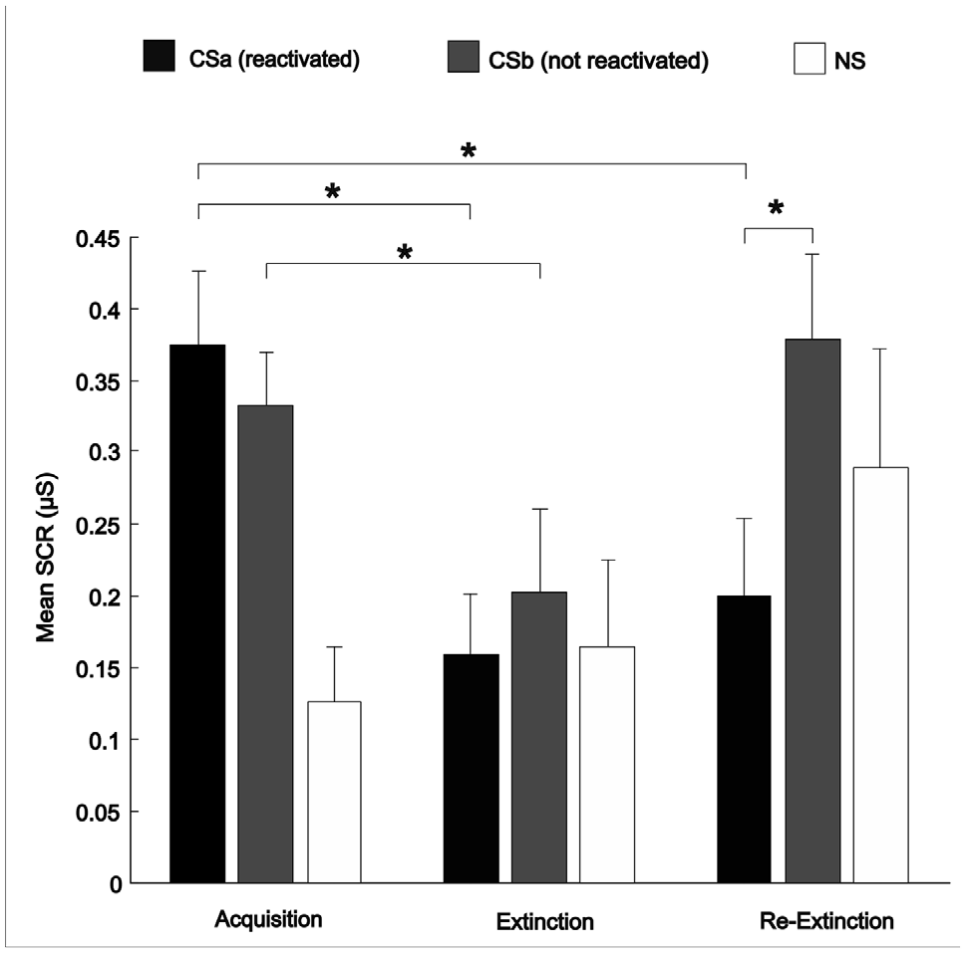
Mean Skin Conductance Response for Acquisition Extinction and Re-Extinction phases. Mean SCRs (reactivated CSa, not reactivated CSb and NS) during Acquisition (mean of the three final trials), Extinction (last trial) and Re-Extinction (first trial). CSs were equally fear conditioned and extinguished. After reinstatement, only CSb showed a significant increment of SCR in Re-Extinction. In contrast, CSa and NS maintained same levels of SCR between Extinction and Re-Extinction. CSa presented a significant reduction of SCR from Acquisition to Re-Extinction. *p<.05. Error bars represent standard errors.

**Figure 3:**
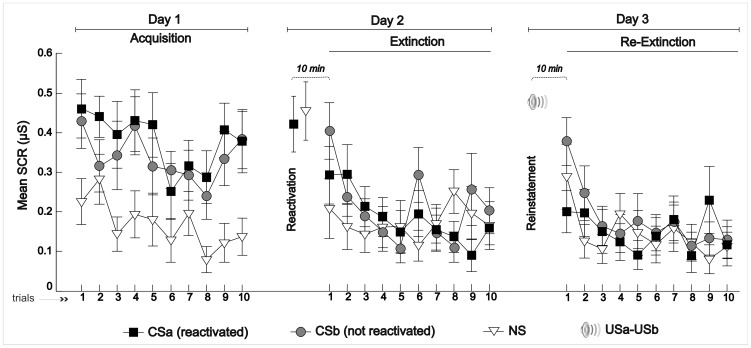
Mean Skin Conductance Response per trials across days. Mean SCRs (reactivated CSa, not reactivated CSb and NS) in non-reinforced trials. CSa and CSb acquired fear conditioning on Acquisition on Day 1. Ten minutes after memory reactivation (of CSa and NS), SCR decreased during Extinction training. On Day 3, ten minutes after reinstatement, CSb recovered fear response in the first trials, whereas CSa maintained equivalent levels of SCR from Extinction to Re-extinction. Error bars represent standard errors.

A paired sample *t*-test between SCR amplitude to all stimuli was performed on Day 1. SCRs to CSa and CSb were equivalent, indicating that both CS generated equal levels of fear conditioning after acquisition on Day 1 (*t* (16) = 1.14, *p* = .273). In contrast, CSa and CSb evoked larger SCRs than the NS condition (*t* (16) = 5.97, *p* = .0001 and *t* (16) = 5.41, *p* = .0001 respectively), pointing out that NS was not associated with an aversive expectation (see Fig. 2). During early acquisition it can be observed (Figure 3) that CSa and CSb show higher SCRs than NS from the very first trial. Note that all values displayed in the figures and used for the analyses represent the trials that were not followed by an US (see Materials and Methods section for stimuli presentation protocol). Thus, the early differences are the result of previous pairings of CS with the aversive stimuli (US) that were sufficient for the participants to learn which stimuli predicted an aversive outcome. Stimuli from all conditions finished the habituation phase with the same SCR response (data not shown).

### Extinction

Next, the decrease of fear response from Acquisition to Extinction was assessed through a series of paired samples *t*-test. Both conditioned stimuli showed significant decrement of fear-induced SCR between Acquisition and Extinction (*t* (16) = 3.49, *p* = .003 for CSa and *t* (16) = 2.48, *p* = .025 for CSb) confirming that fear was successfully extinguished on Day 2 after extinction training. Furthermore, a repeated measures ANOVA with Stimuli (CSa, CSb and NS) as within subject factor for Day 2, demonstrated that SCR of both conditioned stimuli decreased to NS levels (*F* <1). The results mentioned above corroborated that our sample only included subjects that acquired and extinguished fear effectively.

### Fear Recovery

Most importantly, we assessed fear recovery on Day 3 using paired samples *t*-tests. First, we compared SCR to the conditioned stimuli CSa and CSb. In Figure 3 it can be observed that in day 3, after reinstatement, fear recovery to the non-reminded stimulus CSb was significantly greater than to CSa, which was reactivated before extinction training (*t *(16) = −3.41, *p* = .004, for the first trial). In addition, when comparing SCR at the last trial of Day 2 to SCR observed at the first trial of the re-extinction phase (Day 3), only responses to CSb showed an increment in fear response (*t *(16) = −2.07, *p* = .055). In contrast, the SCR to CSa maintained the same response level at Extinction and at Re-extinction (*t *(*1*6) = −0.67, *p* = .51). To further confirm that CSa did not recover fear response while CSb did, we compared the averaged SCR to the last 3 trials of the acquisition phase (Day 1) with the SCR on the first trial of re-extinction. A significant reduction of SCR was observed only for the retrieved CSa (*t* (16) = 3.16, *p* = .006) but not for CSb (*t* (16) = −0.86, *p* = .4) and neither for NS (*t* (16) = −1.68, *p* = .11). Indeed an apparent increment of SCR for NS after reinstatement is observed in Figure 3 but this effect was not significant (*t* (16) = −1.13, *p* = .275, from extinction to re-extinction).

## Discussion

The aim of the current investigation was to replicate Schiller et al. [33] findings in a novel version of the paradigm, using a within-subject design and auditory aversive stimuli instead of an electrical shock. Supporting these previous findings, our results demonstrated that Extinction training conducted 10 minutes after retrieval prevented the reinstatement of fear. The current investigation represents, to the best of our knowledge, the first successful replication of Schiller et al. [33] behavioral manipulation.

These results contrast with the failure in replicating this paradigm by Soeter et al. [28] and Kindt et al. [36]. The reason for this discrepancy may be explained by some methodological differences between these studies and ours (see Figure 1). In both Soeter and Kindt studies [28], [36] it is conceivable that the introduction of additional measurement techniques have rendered the behavioral manipulation less effective. For instance, both studies used fear-relevant pictures that are especially resistant to extinction [38] instead of neutral stimuli (geometric figures) as was the case in ours and in Schiller et al. [33] study. These fear-relevant stimuli, although successfully extinguished on day 2, generated a stronger conditioning [39] making this fear association more resistant to undergo reconsolidation [40]. In line with a stronger conditioning procedure, both Soeter and Kindt studies [28], [36] used larger percentage of parings between CS and US: 100% of the times in the within-subject design [28] and 75% of times in the between-subjects design [36] in comparison with our study and Schiller et al. [33] study (37.5% of the times). This stronger training protocol might have inhibited the induction of the reconsolidation process, as has been described in experiments with rats [41]. For instance, Wang et al. [41] demonstrated that an increment in the number of reinforced stimuli generates a down regulation of the molecular mechanism that triggers reconsolidation in the amygdala making the fear memory transiently resistant to disruption.

In addition, in order to measure the startle reflex response, both Soeter and Kindt studies [28], [36] introduced sounds of 104 db when presenting CS and during intertrial intervals across all experimental phases. Startle stimuli themselves are capable of supporting fear conditioning [42], in consequence it could have been rather difficult to find fear attenuation even if the behavioral manipulation would have succeeded. On the other hand, owing to their intrinsic negative and fearful value, these additional stimuli could have created a more threatening environment, increasing context fear conditioning and thus hindering the restoration of the fearful memory as safe.

On the other hand, the introduction of online-ratings of US expectancy in Kindt and Soeter [36] and online-ratings of distress in Soeter and Kindt [28], encouraged participants to focus their attention in the CSs [36]. This continuous evaluation of the association between CS-US could have overstrengthened the more conscious association between CS-US. This cortical representation of CS-US might have elicited fear responses in the amygdala [43] even if the association would have been effectively disrupted by the behavioral manipulation.

One difference between ours and Schiller et al. [33] and Kindt et al. [36] designs is that we included an initial habituation phase (Kindt and collaborators conducted a habituation but only for the startle probe tone) (Figure 1). This phase was included to establishing an equal baseline for the initial responses to all stimuli prior to the acquisition phase. Although habituation can be considered a learning phase per se, in our case, since there was no gap between habituation and acquisition, it is likely that participants have processed and consolidated both habituation and acquisition as a single phase. Thus, it is improbable that this experimental difference might account for any discrepancy upon studies.

Another point worth mentioning where contrasting results are observed is that Kind and colleges [28], [36] showed a significant increase in the SCR for the NS from extinction to reinstatement whereas this increment was not observed in Schiller et al. [33]. In the current study, an apparent increase can be observed in figure 3 but this increment is not significant. Several reasons can explain an increment in NS after reinstatement. On the one hand, as it has been reported in similar studies, [44]–[47] the context is able to form an association between US an NS without these stimuli being previously paired [45], [48], [49]. Given that after reinstatement the context acquired an aversive value, it is plausible that by mediated conditioning [50] the NS value changes (eliciting fear response) because it had been previously associated with the context [47]. On the other hand, note that in the two studies in which no significant NS increment was found (ours and Schiller et al. 2010, second experiment) NS was reactivated. This safely updating of NS might have impeded a significant increment in both studies. Finally, our hint of NS incremented response might also be explained as the result of a general orienting response since stimuli presentation was fully randomized. Instead, Schiller et al. [33] added a NS before the randomized presentation of stimuli in the re-extinction training to capture the orienting response and remove it afterwards avoiding this effect.

The fact that many factors could prevent the induction of reconsolidation and that the context can become a powerful source of the reinstatement of fear, unveil the constraints and caveats of this behavioral manipulation when having in mind a potential therapeutic application. Indeed, there are critical differences between PTSD fear conditioning and laboratory experiments. In the case of PTSD or phobias, patients present stronger conditioning (due to the presence of stronger and traumatic US), a more complex nature of the CS (i.e. objects, places, social situations, etc, instead of color squares) and fear associations that might have been encoded for a longer time period before being psychologically treated (not only some days). In addition, most of the time patients present multiple conditionings related with the traumatic event and these environmental cues become associated with the event being capable of acting as reminders for the recall of the traumatic experience [51].

Nowadays, many researchers in the reconsolidation field have been faced with memories that due to of their physiological nature resist engaging the reconsolidation process setting the “boundaries of reconsolidation” [52], [53]. For example, it is known that stronger fear memories are particularly resistant to undergo reconsolidation [41]. In this scenery, erasing or updating the memory of a conditioned response that is characterized by such resilience as in PTSD or phobias, leaves us a long way until this method could be applied as a successful therapy in patients [54].

However, the fact that some memories are more resistant to be destabilized, it only means that they are still capable of engaging reconsolidation but under different conditions [41], [51]. Consequently, an improvement of this valuable behavioral manipulation is most needed. Today, there is evidence that reconsolidation is not engaged by being merely retrieved but instead there are specific reactivation conditions for this process to occur [40]. Factors like the structure of the reminder are decisive to trigger reconsolidation [55]; for instance the reminder duration has to be adequate in order to produce reconsolidation and not extinction [56]. Accordingly, the reminder offset should delimit an interval before extinction training [57]. On the other hand, since reconsolidation is a natural adaptive mechanism to update memories, this process is more likely to be activated when reactivation contingencies present relevant novel information worth to be incorporated in the old memory [23], [58], thus in order to induce reconsolidation, the reminder should generate a mismatch between what is expected and what actually happens [57]. In line, demonstrating that resistance to reconsolidation can be transient, Wang et al. [41] showed that strong fear auditory memories that initially did not undergo reconsolidation were able to activate this process after a time when the fear association was transformed into a hippocampus independent memory.

From a research point of view, the fear conditioning paradigm has been recently acknowledged of having ecological validity as a model of anxiety disorders [59]. Our version of the paradigm, with auditory aversive sounds, could be applied to research in patients with Middle Temporal Lobe damage [60], [61] allowing further insight into the neural mechanisms underlying reconsolidation and the structures that might play a decisive role in the induction or prevention of this process.

The fact that Soeter and Kindt (2011) showed disruption of reconsolidation with the administration of propanolol in the same experimental design where they failed to reproduce Schiller et al. (2010) behavioral manipulation speaks about the strong inhibitory effects that this drug exerts on reconsolidation in the amygdala. However, it is still unclear why SCR recovery is not prevented in their experiments (in contrast with the successful Startle Reflex results). This issue is critical if we consider that hyper-vigilance is one of the primary PTSD symptoms due to an over-excited noradrenergic system [62], [63] an essential component of the sympathetic system that drives the SCR measured in most fear conditioning paradigms.

In summary, even though our results are encouraging, the fact that small differences in the protocol resulted in deviant results in previous studies (Kindt and Soeter, 2011) shows that this behavioral manipulation is not robust enough to be translated into clinical application yet. To surmount these limitations, greater research is required to determine the optimal reactivation conditions under which these strong and resilient fearful memories would undergo reconsolidation and hence be successfully disrupted. Our novel version of Schiller et al. [33] paradigm, represents an important step in the long way to discover an efficient and safe mechanism to erase maladaptive fearful memories.

## Materials and Methods

### Participants

#### Ethics statement

The study was approved by the ethics committee of the University of Barcelona and all participants signed a written informed consent before enrolling in the experiment.

Twenty-one healthy participants (7 males and 14 females) were recruited at the University of Barcelona by email advertisement, the mean age was 23.4 (SD = 5.11). All participants reported no history of psychiatric or neurological disease. All participants were remunerated for their participation at the end of each experimental day. Four participants were excluded from statistical analysis because they did not fulfill the criterion for acquisition and extinction *(see criterion for acquisition and extinction section).*

### Stimuli and Procedure

Three different colour squares (yellow, pink and blue) of 5×5 cm were used; two of them served as conditioned stimuli (CSa and CSb) and the other one served as the neutral stimulus (NS). Each square was presented for 4 seconds. In all experimental phases, inter-trial intervals varied between 8 seconds and 10 seconds from the offset of the last visual stimulus to the onset of the following. The inter-trial interval was managed by the researcher, so that the next trial did not start until the SCR reached baseline levels after each stimulus presentation.

Two different loud shrill sounds were used as the aversive stimulus (USa and USb): a girl screaming and a pig squealing. Sounds were set at 98 db and 2.4 seconds length the former, and 96 db and 1.7seconds length the latter. Both sounds co-terminated with the visual stimuli. The contingencies between squares and sounds were counterbalanced across participants.

Visual stimuli were presented over a black background on a nineteen inch computer monitor, squares were placed over a 15×11 cm white rectangle. The auditory stimuli were delivered through loudspeakers located symmetrically at each side of the screen. Stimuli presentation was implemented using the E-Prime software. Participants were tested in an electrically isolated, dimly lighted and sound attenuated booth, and they were monitorized through a camera over the entire session.

### Phases of the Experiment

The experiment was conducted during three consecutive days with a 24 hrs interval (experimental design is summarized in Table 1).

### Day 1

#### Habituation phase

In order to reach a SCR baseline to the appearance of the squares, the visual stimuli were presented repeatedly to the participants in random order six times each.

#### Fear acquisition phase

Immediately after habituation, CSa and CSb were presented 10 times non-reinforced and 6 times co-terminating with its aversive sound (USa and USb). The neutral stimulus (NS) was presented 10 times, never paired with a sound, overall using the same proportion as in Schiller et al. [33]. The order of presentations of the trials was pseudo-randomized, so that reinforced stimuli were distributed early across the session. In addition, we made sure that at least one presentation of CS-US occurred before its corresponding CS (alone).

Participants were instructed to pay attention to the computer screen and try predicting the aversive sounds that would be elicited after the appearance of two of the three squares.

### Day 2

#### Memory reactivation

A single presentation of CSa (without US) and NS but not CSb was used to reactivate each memory episode. After these presentations, participants watched a ten minutes TV show [35].

#### Extinction

Immediately after that, participants underwent extinction training in which the NS and the conditioned stimuli (both CSa and CSb) without the US were presented ten times each. The order of presentations of the trials was randomized.

### Day 3

#### Reinstatement

Subjects received 4 unsignaled USa and 4 unsignaled USb. The order of presentations of trials was randomized. Afterwards, participants played a reposed computer based card or skill game for ten minutes (i.e. Solitaire, Minesweeper).

#### Re-extinction

In order to assess fear recovery, participants underwent re-extinction training. Thus, subjects were presented with 10 presentations of each conditioned stimulus (CSa and CSb) and 10 presentations of the NS. The order of presentations of the trials was randomized.

### Data Analysis

The statistical analysis was made with SPSS. We performed repeated measure Analysis of Variance and Paired Sample T-Test for the following analysis between conditions.

### Selection of Trials

To statistically test our predictions, we used the averaged SCR amplitude modulation to the last three trials at the Acquisition phase, the SCR amplitude to the last trial in the Extinction training and the SCR amplitude to the first trial in the re-extinction phase [33].

### SCR Assessment

While performing the tasks, SCR was recorded using two Ag-AgCl electrodes, to a Brainvision Brainamp device. The electrodes were attached to the forefinger and the middle finger of the left hand and placed between the first and second phalanges. SCR recordings were filtered using a low pass filter of 1 Hz before been analyzed with Matlab 7.7.

Fear was indirectly measured using the SCR as a reliable index of expectation [64] and fear [1]. To assess fear separately from the unconditioned responses to the aversive sounds, we included only non-reinforced trials of CS in the analysis. The level of SCR was determined by taking the base-to-peak difference for the first waveform in the 0.5 s –6.5 s window after stimulus onset. The resulting SCR amplitude value was normalized to the SCR amplitude of the baseline period (averaged over −200 ms to stimuli onset) and then squared-root transformed to fulfill the parameters of a normal distribution [27], [28].

### Criterion for Acquisition and Extinction

Because fear recovery could not be assessed if fear responses were not successfully acquired and/or extinguished, participants were not included in statistical analysis if they did not acquire fear conditioning on day 1 or if they did not extinguish fear response on day 2, to both of the conditioned stimuli (CSa and/or CSb). The exclusion criteria were based on the SCR values obtained in response to CS (a and/or b) in the last third of acquisition (three last trials) and in the last trial of extinction. That is, participants were excluded if during the final trials of acquisition, the SCR value in response to NS was equal or greater than SCR values to any of the CS. They were also excluded in case that SCR to any CS in the extinction phase (measure in the last trial) was greater than their SCR averaged value during the last 3 trials at the acquisition phase.

Seventeen of the twenty-one subjects enrolled in this study met the specified acquisition and extinction inclusion criteria and were thus included in the analyses.
